# Automethylation Activities within the Mixed Lineage Leukemia-1 (MLL1) Core Complex Reveal Evidence Supporting a “Two-active Site” Model for Multiple Histone H3 Lysine 4 Methylation*

**DOI:** 10.1074/jbc.M113.501064

**Published:** 2013-11-14

**Authors:** Anamika Patel, Valarie E. Vought, Stephen Swatkoski, Susan Viggiano, Benny Howard, Venkatasubramanian Dharmarajan, Kelsey E. Monteith, Gillian Kupakuwana, Kevin E. Namitz, Stephen A. Shinsky, Robert J. Cotter, Michael S. Cosgrove

**Affiliations:** From the ‡ Department of Biochemistry and Molecular Biology, State University of New York Upstate Medical University, Syracuse, New York 13210,; the § Department of Biology, Syracuse University, Syracuse, New York 13244, and; the ¶ Department of Pharmacology and Molecular Sciences, The Johns Hopkins School of Medicine, Baltimore, Maryland 21202

**Keywords:** Chromatin Histone Modification, Enzyme Mechanisms, Epigenetics, Histone Methylation, Leukemia, Automethylation, Self-methylation

## Abstract

The mixed lineage leukemia-1 (MLL1) core complex predominantly catalyzes mono- and dimethylation of histone H3 at lysine 4 (H3K4) and is frequently altered in aggressive acute leukemias. The molecular mechanisms that account for conversion of mono- to dimethyl H3K4 (H3K4me1,2) are not well understood. In this investigation, we report that the suppressor of variegation, enhancer of zeste, trithorax (SET) domains from human MLL1 and *Drosophila* Trithorax undergo robust intramolecular automethylation reactions at an evolutionarily conserved cysteine residue in the active site, which is inhibited by unmodified histone H3. The location of the automethylation in the SET-I subdomain indicates that the MLL1 SET domain possesses significantly more conformational plasticity in solution than suggested by its crystal structure. We also report that MLL1 methylates Ash2L in the absence of histone H3, but only when assembled within a complex with WDR5 and RbBP5, suggesting a restraint for the architectural arrangement of subunits within the complex. Using MLL1 and Ash2L automethylation reactions as probes for histone binding, we observed that both automethylation reactions are significantly inhibited by stoichiometric amounts of unmethylated histone H3, but not by histones previously mono-, di-, or trimethylated at H3K4. These results suggest that the H3K4me1 intermediate does not significantly bind to the MLL1 SET domain during the dimethylation reaction. Consistent with this hypothesis, we demonstrate that the MLL1 core complex assembled with a catalytically inactive SET domain variant preferentially catalyzes H3K4 dimethylation using the H3K4me1 substrate. Taken together, these results are consistent with a “two-active site” model for multiple H3K4 methylation by the MLL1 core complex.

## Teaser

**Background:** The MLL1 core complex mono- and dimethylates histone H3 lysine 4 (H3K4).

**Results:** MLL1 automethylates a conserved cysteine residue in its active site cleft.

**Conclusion:** MLL1 automethylation is inhibited by unmodified histone H3 but not by histones previously mono-, di-, or trimethylated at H3K4.

**Significance:** The pattern of automethylation inhibition is consistent with distinct active sites for mono- and dimethylation of H3K4.

## Introduction

Human mixed lineage leukemia protein-1 (MLL1, KMT2A, EC 2.1.1.43) is a member of the SET1 family of histone H3 lysine 4 (H3K4) methyltransferases and is frequently altered in acute myeloid and lymphoid leukemias (1–4). The MLL1 protein contains an evolutionarily conserved suppressor of variegation, enhancer of zeste, trithorax (SET) domain that predominantly catalyzes monomethylation of H3K4 (5), a reaction that is required for subsequent H3K4 di- and trimethylation and transcriptional activation in eukaryotes (6–10). Although the mechanisms that regulate the degree of H3K4 methylation are not well understood, H3K4 di- and trimethylation by SET1 family enzymes are known to require a sub-complex of proteins called WRAD, which consists of WDR5 (tryptophan-aspartate repeat protein-5), RbBP5 (retinoblastoma-binding protein-5), Ash2L (Absent-small-homeotic-2-like), and DPY-30 (Dumpy-30) (6, 11). A central question is to understand how the degree of H3K4 methylation is regulated by MLL1 and WRAD, together forming what is known as the MLL1 core complex.

Two models for regulation of the degree of H3K4 methylation by the MLL1 core complex have emerged. In the two-active site model, H3K4 mono- and dimethylation are sequentially catalyzed in a stepwise manner at two distinct active sites within the complex. This model is supported by the recent demonstration that WRAD possesses H3K4 di-methyltransferase activity within the complex that is independent of the catalytic activity of the MLL1 SET domain (5). In the one-active site model, H3K4 mono-, di-, and trimethylation (H3K4me1, 2, or 3) are catalyzed in the same active site of the MLL1 SET domain, whose conformation is allosterically regulated by interaction with WRAD. The one-active site model is inferred from amino acid sequence alignments suggesting that the MLL1 SET domain requires a conformational change to increase the active site volume and accommodate multiple methylation events. Cited in support of this hypothesis is the crystal structure of the MLL1 SET domain, which, contrary to other SET domain structures, is locked in an inactive “open” conformation in the presence and absence of ligands (12). It has been suggested that interaction with WRAD is required to induce the domain closure necessary for full catalytic activity (12). However, an analysis of crystal packing of the x-ray crystal structure suggests that the open conformation of the MLL1 SET domain may be constrained by unnatural crystal contacts (13). Therefore, the conformational dynamics of the MLL1 SET domain in solution remain an open question. In addition, although it has been demonstrated that the MLL1 SET domain has inherent monomethyltransferase activity (5), the discovery that WRAD has intrinsic H3K4 methyltransferase activity (5, 14) raises the question as to whether the SET domain of MLL1 catalyzes H3K4 di- or trimethylation within the complex.

In this investigation, we report on a relatively robust automethylation reaction in the MLL1 SET domain and demonstrate that it serves as a convenient probe for histone and cofactor binding. We show that a conserved cysteine residue (Cys-3882 in MLL1) in the SET domain active sites of human MLL1 and its *Drosophila melanogaster* ortholog Trithorax (Cys-3641) is methylated in an intramolecular manner, which is inhibited by unmodified histone H3. The location of the automethylation in the SET-I subdomain suggests that the MLL1 SET domain possesses significantly more conformational plasticity in solution than suggested by its crystal structure. We also report that MLL1 methylates Ash2L, but only when assembled within the MLL1 core complex in the absence of histone H3, suggesting a restraint for the quaternary structure of the complex. Using MLL1 and Ash2L automethylation reactions as probes for histone binding within the MLL1 core complex, we demonstrate that stoichiometric amounts of full-length unmethylated histone H3, and to a lesser extent unmethylated histone H3 peptides, significantly inhibit MLL1 automethylation and Ash2L methylation reactions. In contrast, despite being a robust substrate for dimethylation, H3K4 monomethylated species do not significantly inhibit MLL1 or Ash2L automethylation reactions, suggesting that they do not bind to the SET domain within the MLL1 core complex during the dimethylation reaction. Supporting this hypothesis, we demonstrate that the H3K4me1 species is preferentially methylated by WRAD when assembled with a catalytically inactive MLL1 SET domain variant. These results are consistent with the two-active site model for multiple lysine methylation. Taken together, we demonstrate that MLL1 and Ash2L automethylation reactions are useful probes for subunit conformational dynamics, substrate and coenzyme binding, complex assembly, and the mechanism of multiple lysine methylation by the human MLL1 core complex.

## EXPERIMENTAL PROCEDURES

### Protein Expression and Purification

Human MLL1 constructs consisting of amino acid residues 3811–3969 (MLL^3811^) and 3745–3969 (MLL^3745^) were expressed in *Escherichia coli* as MBP^7^ or GST fusion proteins, respectively, and purified as described previously (15). Full-length human WDR5, RbBP5, Ash2L, and DPY-30 cDNAs were expressed individually in *E. coli* as N-terminal His_6_ fusion proteins and purified as described previously (5, 15). Aliquots of each protein were maintained at −80 °C until thawed and used directly in assays.

### Mutagenesis and Histones

Point mutations were introduced using the QuikChange II XL site-directed mutagenesis kit (Agilent Technologies). Plasmids were sequenced to ensure the presence of the intended mutations and the absence of unintended mutations. Unmodified and modified histone H3 peptides (residues 1–20) with C-terminal GGK(biotin) were synthesized from Global Peptide. Full-length unmodified and H3K4 mono-, di-, and trimethylated histone H3 proteins as methylated lysine analogs (MLA) were purchased from Active Motif.

### Methyltransferase Assays

Radiolabeling assays were conducted by combining 7 μg of MLL^3811^ or MLL^3745^ with 1 μCi of [^3^H]methyl-*S*-adenosylmethionine ([^3^H]AdoMet) in the absence or presence of 300 μm histone H3 peptide or 4 μg of full-length unmodified or methylated histone H3 proteins (Active Motif) in reaction buffer (50 mm Tris, pH 8.5, 200 mm NaCl, 3 mm dithiothreitol, 5 mm MgCl_2_ and 5% glycerol). Control experiments revealed that the MLL1 SET domain rapidly inactivates at temperatures above 20 °C. The reactions were therefore incubated at 15 °C for 4 or 8 h and quenched by the addition of 1× SDS loading buffer. The samples were separated by 4–12% SDS-PAGE and stained with Coomassie Brilliant Blue. Gels were then soaked in autoradiography enhancer solution (Enlightening, PerkinElmer Life Sciences), dried, and exposed to film at −80 °C for 24 or 48 h. For quantitative measurements, bands corresponding in size to MLL1 or histone H3 were excised after Coomassie staining, dissolved in Solvable (PerkinElmer Life Sciences), and counted by liquid scintillation counting.

### Concentration Dependence of Automethylation

For MLL1 and MLL1 core complex concentration dependence assays, various concentrations of enzyme (1–30 μm) were incubated with [^3^H]AdoMet (50 μm) in reaction buffer for 4 h. Samples were quenched with 1× SDS loading buffer at various time points and separated by 4–12% SDS-PAGE and stained with Coomassie Brilliant Blue. Gels were soaked in autoradiography enhancer (Enlightening, PerkinElmer Life Sciences) solution, dried, and exposed to film for 1–3 weeks at −80 °C. Multiple exposures were taken at different intervals to ensure that adequate signal-to-noise could be obtained without band saturation. Films were scanned with the Bio-Rad ChemiDoc^TM^ MP system, and bands for each time course were quantitated by densitometry using Bio-Rad Image Lab^TM^ software or ImageJ software (16). Relative intensity values were plotted using GraphPad Prism 6 (GraphPad Software Inc.) and fitted with linear regression to determine methylation rate. Methylation rate *versus* enzyme concentration was plotted in a log-log plot and fitted with linear regression to determine reaction order.

### Apparent K*_m_* Value Determination

To determine the apparent *K_m_* value for MLL1 automethylation, [^3^H]AdoMet concentration was varied (0–20 μm) with a constant concentration of MLL^3745^ (15 μm) for 3 h. Samples were quenched at various time points with 1× SDS loading buffer, separated by 4–12% SDS-PAGE, and stained with Coomassie Brilliant Blue. Gels were soaked in autoradiography enhancer (Enlightening, PerkinElmer Life Sciences) solution, dried, and exposed to film for 7 days at −80 °C. Densitometry of the fluorogram bands was conducted with ImageJ. Methylation rates were plotted as a function of AdoMet concentration and fitted by nonlinear least squares regression to the Michaelis-Menten Equation 1. To determine the apparent *K_m_* value for AdoMet in the histone H3 methylation reaction, 7 μm MLL1 was incubated with different concentrations of [^3^H]AdoMet (0–100 μm) at a fixed concentration of histone H3 peptide (500 μm) consisting of residues 1–20 followed by a GGK-biotin on the C terminus. Reactions were quenched by the addition of EDTA (0.5 m) at various time points. H3 peptides were pulled down with streptavidin-agarose and washed three times in phosphate-buffered saline with Tween 20 (PBST). Beads were then counted by liquid scintillation counting. Methylation rates were plotted as a function of AdoMet concentration and fitted to the Michaelis-Menten Equation 1 as described above.

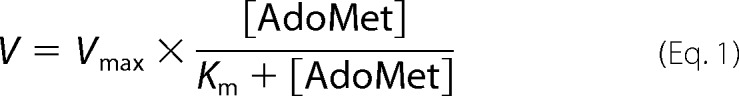


### Mass Spectrometry

#### 

##### Sample Preparation

Purified MLL^3811^ and MLL^3745^ proteins at a concentration of 0.8 mg/ml were incubated with 1 mm AdoMet at 15 °C for 18 h. Samples were diluted 5-fold and subjected to overnight trypsin digestion at 37 °C (1:20 enzyme/substrate ratio). The samples were not reduced or alkylated prior to digestion. Digestions were terminated by the addition of 1.0% TFA.

##### MALDI-TOF Mass Spectrometry

MALDI-TOF mass spectrometry was carried out on a Kratos Axima TOF^2^ mass spectrometer (Shimadzu, Manchester, UK). One microliter of the digested samples was applied to the sample plate and allowed to air dry. This was followed by addition of 1 μl of 10 mg/ml α-cyano-4-hydroxycinnamic acid matrix solution to the dried samples, which was again allowed to air dry. Spectra were acquired in reflectron mode as an average of 100 profiles.

##### ESI-HPLC Mass Spectrometry

The tryptic digestion products from MLL^3745-WT^ were separated and analyzed by nESI LC MS/MS on a hybrid LTQ-Orbitrap mass spectrometer (Thermo, San Jose, CA). A 4.0-μl aliquot of the digested sample was first diluted 10-fold with 0.1% formic acid. A 5.0-μl aliquot of the peptide mixture was then injected onto a C_18_ reverse phase column and gradient eluted with acetonitrile, 0.1% formic acid over 50 min. Full MS scans were carried out in the Orbitrap, and all fragment ions were measured in the LTQ. Precursors were selected in data-dependent mode and fragmented using collision-induced dissociation.

## RESULTS

### 

#### 

##### MLL1 SET Domain Possesses a Robust and Irreversible Automethylation Activity

**FIGURE 1.**
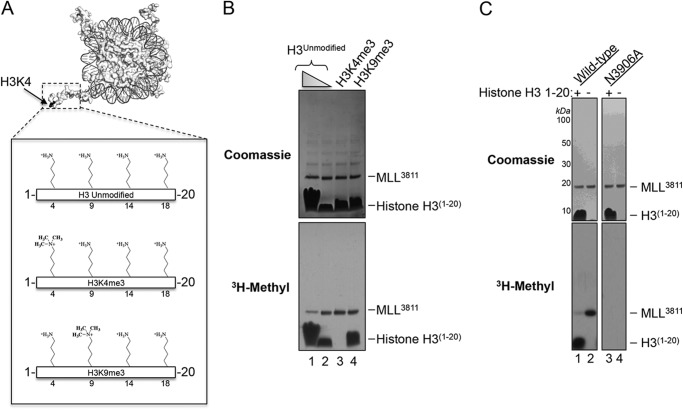
**MLL1 SET domain undergoes an automethylation reaction that is inhibited by histone H3.** *A,* surface representation of the nucleosome core particle (PDB code 1KX5 (61)) is shown with the position of H3K4 indicated. The *dashed box* encompasses residues 1–20 of histone H3. The *box below* shows a schematic of histone H3 peptides used in this investigation, consisting of amino acid residues 1–20. The positions of lysine or trimethyl-lysine residues are indicated. *B,* comparison of enzymatic activity of the MLL1 SET domain (amino acid residues 3811–3969, MLL^3811^) among histone H3 peptides that were unmodified (H3^Unmodified^, *lanes 1* and *2*) or previously trimethylated at Lys-4 (H3K4me3, *lane 3*), or trimethylated at Lys-9 (H3K9me3, *lane 4*). *C,* MLL1 automethylation is abolished when Asn-3906 is replaced with alanine.

Previous studies revealed that the SET1 family of SET domain proteins specifically methylates lysine 4 of histone H3 (7, 17–20). We cloned, expressed, and purified an enzymatically active human MLL1 SET domain fragment consisting of amino acid residues 3811–3969 (MLL^3811^) and compared its enzymatic activity with a group of synthetic histone H3 peptides that mimic the histone H3 N-terminal tail (residues 1–20). These peptides were either unmethylated or previously trimethylated at lysine 4 (H3K4me3) or lysine 9 (H3K9me3) (Fig. 1*A*). We observed that MLL^3811^ methylated unmodified and H3K9me3 peptides (Fig. 1*B*, *lanes 1, 2,* and *4*) but did not methylate the H3K4me3 peptide (Fig. 1*B*, *lane 3*). Because trimethylation of lysine 4 prevents methylation in H3 peptides containing a total of four lysine residues (4, 9, 14, and 18) (Fig. 1*A*), these results suggest that MLL^3811^ has a high specificity for lysine 4 of histone H3.

Unexpectedly, in our methylation assays with H3 peptides, we also observed robust automethylation of MLL^3811^ (Fig. 1*B*). The amount of automethylation appeared to be dependent on the concentration of histone peptide, as automethylation was reduced with excess unmethylated histone H3 (Fig. 1*B*, *lane 1*). To rule out the possibility that MLL1 is methylated by a contaminating methyltransferase from *E. coli*, we created an MLL1^3811^ variant with a point mutation (MLL^3811(N3906A)^) that abolishes its enzymatic activity and tested for radioactive labeling after incubation with [^3^H]AdoMet. We previously demonstrated that asparagine 3906 of MLL1 is required for binding the methyl donor AdoMet but not for the overall structure of MLL1 (5). When Asn-3906 of MLL^3811^ was replaced with alanine, both histone H3 methylation and MLL1 automethylation reactions were abolished (Fig. 1*C*). These results indicate that MLL1 automethylation depends on the activity of the MLL1 SET domain, ruling out the possibility that MLL1 is methylated by a contaminating methyltransferase. Similar results were observed with an MLL1 construct containing amino acid residues 3745–3969 (MLL^3745^) (see below).

**FIGURE 2.**
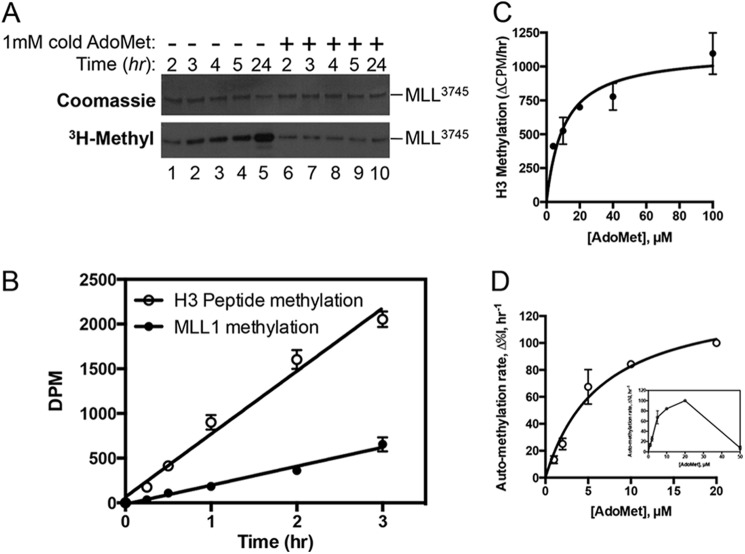
**MLL1 automethylation is irreversible and relatively robust.** *A,* MLL^3745^ was incubated with 1 μm [^3^H]AdoMet for 2 h, followed by the addition of 1 mm unlabeled (cold) AdoMet or an equivalent volume of reaction buffer. Reactions were quenched at the indicated time points with 1× SDS loading buffer and separated by SDS-PAGE. *Upper panel* shows Coomassie Blue-stained gel, and *lower panel* shows the fluorogram of the same gel. *B,* 5 μm MLL^3745^ was incubated with 25 μm [^3^H]AdoMet in the presence or absence of 250 μm histone H3 peptide (residues 1–20). Reactions were quenched at the indicated time points with 1× SDS loading buffer and separated by SDS-PAGE. Histone peptide and MLL^3811^ bands at the different time points were excised from the gels and quantitated by liquid scintillation counting. *Open circles* show the rate of histone H3 peptide methylation, and *closed circles* show the rate of MLL^3811^ automethylation in the absence of histone H3 peptide. Linear regressions gave slopes of 704 ± 46 (*R*^2^ = 0.98) and 212 ± 12 (*R*^2^ = 0.99) for histone H3 and MLL^3811^ automethylation reactions, respectively. *C,* apparent *K_m_* determination for AdoMet in the histone H3 methylation reaction catalyzed by MLL1. The change in counts from LSC (Δcpm, h^−1^) *versus* AdoMet concentration was fit with nonlinear least squares regression to the Michaelis-Menten equation with an apparent *K_m_* value for AdoMet of 10.4 ± 3.1. *D,* apparent *K_m_* determination for AdoMet in the MLL1 automethylation reaction. Changes in relative intensity per h (Δ%*I*, h^−1^) were plotted against AdoMet concentration (0–20 μm) and fit with the Michaelis-Menten equation with an apparent *K_m_* value of 6.5 ± 1.5. *Inset* shows automethylation activity over the concentration range 0–50 μm, which displays a pattern of substrate inhibition at 50 μm AdoMet.

To determine whether MLL1 automethylation is reversible, we preincubated the enzyme with 1 μm [^3^H]AdoMet for 2 h and compared automethylation over time after the addition of 1 mm cold (nonradioactive) AdoMet or an equivalent volume of buffer (Fig. 2*A*). The results show little change in the incorporation of [^3^H]methyl during the incubation with cold AdoMet (Fig. 2*A, lanes 6–10*) as compared with the incorporation observed after the addition of buffer (*lanes 1–5*), suggesting that the reaction is irreversible under these conditions. In addition, enzymatic assays show that the rate of automethylation in the absence of histone H3 peptide is ∼⅓ that of the rate of histone H3 methylation at a substrate concentration of 250 μm (Fig. 2*B*). These results indicate that the rate of automethylation in the absence of histone H3 is relatively robust. We also estimated from radiolabeling that ∼3% of the MLL1 sample is methylated under these conditions after 22 h. This low level of [^3^H]methyl incorporation may be indicative of the low catalytic rate of automethylation or of the possibility that the majority of enzyme is purified from *E. coli* in a highly automethylated form.

We next compared the apparent *K_m_* values for AdoMet between MLL1 automethylation and histone H3 methylation reactions. For the histone H3 methylation reaction with a fixed concentration of histone H3 peptide (500 μm), the apparent *K_m_* value for AdoMet was 10.4 ± 3.1 μm (Fig. 2*C*). The apparent *K_m_* value for AdoMet in the MLL1 automethylation reaction in the absence of histone H3 was similar at 6.5 ± 1.5 μm, when assayed over the concentration range of 0–20 μm AdoMet (Fig. 2*D*). The results suggest that AdoMet binding is likely to be similar in both the MLL1-catalyzed automethylation and histone H3 methylation reactions. However, unlike the histone H3 methylation reaction, the MLL1 automethylation reaction exhibited a pattern of partial substrate inhibition at a higher concentration of AdoMet (50 μm) (Fig. 2*D*, *inset*). This may indicate the presence of a second AdoMet-binding site that diminishes the MLL1 automethylation reaction but not the histone H3 methylation reaction.

##### MLL1 Automethylation Is Intramolecular

**FIGURE 3.**
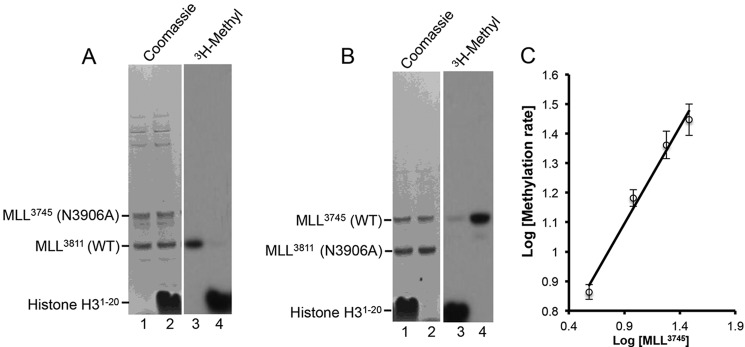
**MLL1 automethylates in an intramolecular fashion.** *A,* wild type MLL^3811^ was incubated with the larger catalytically inactive N3906A MLL^3745^ variant and 1 μm [^3^H]AdoMet in the absence (*lanes 1* and *3*) or presence of histone H3 peptide (*lanes 2* and *4*). Reactions were quenched after 4 h and separated by SDS-PAGE. *Left panel* shows Coomassie Blue-stained gel, and *right panel* shows the fluorogram of the same gel. *B,* same as in *A* except that wild type MLL^3745^ was incubated with the catalytically inactive N3906A MLL^3811^ variant in the absence (*lanes 1* and *3*) and presence of histone H3 peptide (*lanes 2* and *4*). *C,* log-log plot showing the concentration dependence of MLL1 automethylation velocity. The data were fit with linear regression showing a slope of 0.7 (*R*^2^ = 0.99). *Error bars* represent one standard deviation from the mean from two independent experiments.

To determine whether MLL1 automethylation occurs in an intra- or intermolecular fashion, we determined whether the wild type MLL^3811^ construct (18 kDa) could methylate a longer MLL1 construct containing residues 3745–3969 (MLL^3745^, 26 kDa) that was made catalytically inactive by replacement of Asn-N3906 with alanine (MLL^3745(N3906A)^). When wild type MLL^3811^ was mixed with the catalytically inactive MLL^3745(N3906A)^ enzyme, no *trans*-methylation of the longer MLL1 construct could be observed, despite significant *cis*-methylation of the wild type MLL^3811^ protein in the absence of histone H3 peptide (Fig. 3*A*, *lane 3*). Similarly, when wild type MLL^3745^ was incubated with the catalytically inactive MLL^3811(N3906A)^, no *trans*-methylation of MLL^3811^ was observed despite robust *cis-*methylation of the longer MLL^3745^ construct (Fig. 3*B*, *lane 4*). These results are consistent with intramolecular automethylation.

To further test this hypothesis, we examined the concentration dependence of MLL1 automethylation. If MLL1 automethylation is intramolecular, then the reaction rate will be first order with respect to MLL^3745^ concentration. In contrast, intermolecular automethylation reactions will be second order or higher. To distinguish these possibilities, we measured the rate of MLL1 automethylation as a function of time and MLL^3745^ concentration. The logarithmic plot of the rate of automethylation as a function of MLL^3745^ concentration shows a slope of ∼0.7 (Fig. 3*C*), which approximates a first order reaction mechanism. Together, these results indicate that the MLL1 automethylation reaction is intramolecular.

##### MLL1 Automethylates a Cysteine Residue in the SET Domain Active Site

**FIGURE 4.**
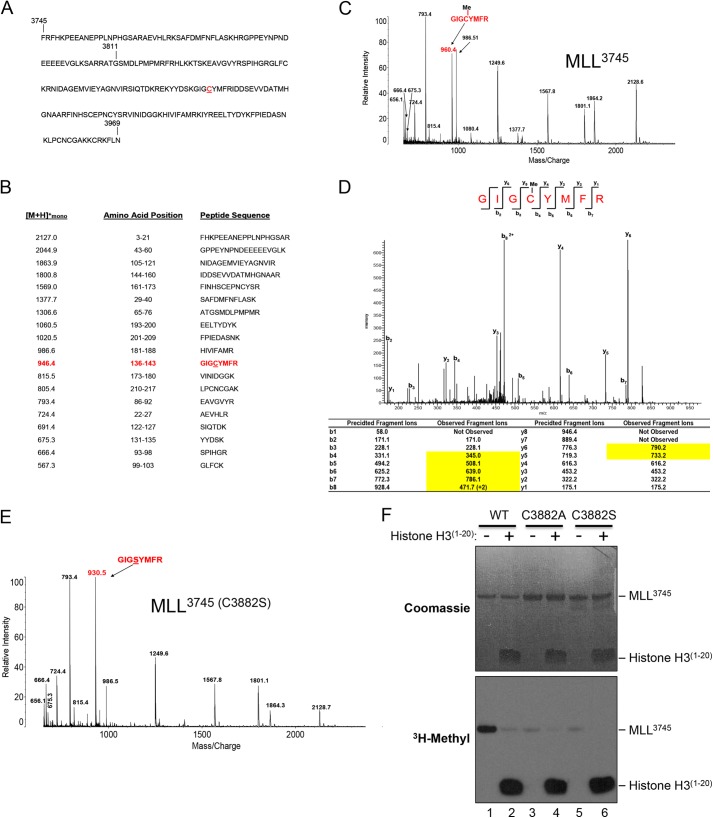
**Mass spectrometry identifies Cys-3882 as a site for MLL1 automethylation.** *A,* sequence of the MLL^3745^ construct; *B, table* of predicted trypsin proteolytic peptides and expected masses. *C,* MALDI-TOF spectrum of the trypsinized MLL^3745^ protein after preincubation with 1 mm AdoMet. The peptide at *m*/*z* 960.4 is indicated and is 14 Da larger than the expected GIGCYMFR peptide at 946.4. *D,* ESI LC MS/MS spectrum of the doubly charged precursor at *m*/*z* = 480.8, which corresponds to the methylated peptide GIGCYMFR. The *table below* displays all predicted and observed b- and y-ions for the unmodified and modified forms of the peptide, respectively. Each of the peptides highlighted in yellow are 14 Da greater than predicted, indicating that Cys-3882 is methylated. *E,* MALDI-TOF spectrum of the trypsinized C3882S MLL^3745^ protein. The peak corresponding to the GIGSYFMR peptide is indicated at *m*/*z* 930.5. *F,* comparison of histone methylation and automethylation activities of wild type and C3882A/S MLL^3745^ proteins in the presence and absence of histone H3 peptide. Reactions were quenched at 8 h and separated by 4–12% BisTris PAGE and visualized by Coomassie Blue staining (*upper panel*) and fluorography (*lower panel*).

Automethylation of MLL1 is surprising given its high specificity for lysine 4 of histone H3 and the lack of lysine 4-like sequences in the primary structure of MLL^3745^ (Fig. 4*A*). To identify the site(s) of automethylation in MLL1, we compared MALDI-TOF mass spectra of tryptic digests of automethylated MLL^3811^ and MLL^3745^ constructs to those of theoretical tryptic peptide sequences (Fig. 4*B*). For MLL^3745^, 12 of the peptide products observed in the mass spectra corresponded to peptide masses generated via *in silico* trypsin digestion. Although a peptide signal corresponding to the theoretical peptide, GIGCYMFR ([M + H]^+^ = 946.4), was not detected, a peptide was observed at *m*/*z* 960.4 that corresponded to a monomethylated form of this peptide (Fig. 4*C*). A similar peptide was observed at *m*/*z* = 960.4 in the mass spectrum of the tryptic digestion products of the MLL^3811^ protein (data not shown). To identify the residue methylated in this peptide, we analyzed digestion products using ESI LC-MS/MS on an LTQ-Orbitrap mass spectrometer. The MS/MS spectrum of the doubly charged precursor at *m*/*z* = 480.8 corresponding to the methylated peptide GIGCYMFR is shown in Fig. 4*D*. Seven b- and six y-ions were identified in the MS/MS spectra that matched predicted fragment ions. The *table* in Fig. 4*D* displays all of the predicted b- and y-ions for the unmodified form of this peptide. Five b-ions (b_4_–b_8_) and two y-ions (y_5_ and y_6_), with *m*/*z* values 14 Da greater than the predicted masses, were observed, which confirmed that this peptide was methylated. Furthermore, in the observed b-ion series, b_2_ and b_3_ had *m*/*z* values that were consistent with their predicted masses. This fragmentation pattern indicates that the site of methylation is Cys-3882.

To confirm that Cys-3882 of MLL1 is a site for automethylation, we replaced Cys-3882 with serine by site-directed mutagenesis and performed MALDI-TOF mass spectrometry on a tryptic digest of the C3882S MLL^3745^ protein that was preincubated with AdoMet (Fig. 4*E*). The only difference observed was a shift in mass of the peak at *m*/*z* = 960.4 in the MLL^3745^-WT spectrum to *m*/*z* = 930.5 in the spectrum of the MLL^3745^ (C3882S) digest (Fig. 4*E*). The peptide at *m*/*z* = 930.5 corresponds to the peptide GIGSYMFR, which contains the mutation site, residue 3882.

To further confirm that Cys-3882 is a site for automethylation, we incubated wild type, C3882S, and C3882A MLL^3745^ SET domain proteins with [^3^H]AdoMet and compared their ability to automethylate by fluorography. The results show that replacement of Cys-3882 with alanine or serine abolishes most, but not all, MLL1 SET domain automethylation (Fig. 4*F*). Analysis of the C3882S MALDI-TOF spectrum did not reveal the identity of the minor automethylation site, which may be due to its low methylation level or it may occur in a peptide fragment that is missing in the sequence coverage. Taken together, these results indicate that Cys-3882 of MLL1 is a major site of automethylation.

##### Cys-3882 Is Located in the SET-I Region of the MLL1 SET Domain

**FIGURE 5.**
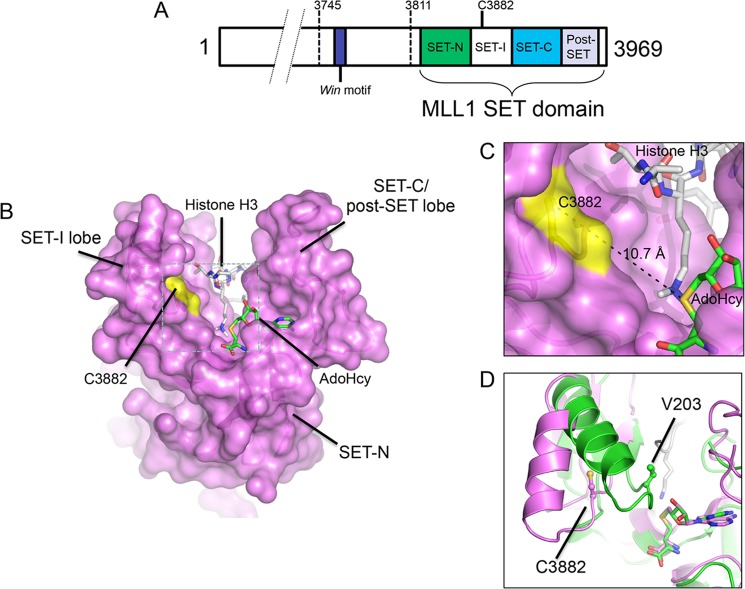
**Cysteine 3882 is located in the SET-I subdomain of MLL1.** *A,* schematic representation of the C-terminal SET domain and Win motif of MLL1. The position of Cys-3882 in the SET-I subdomain is indicated. The SET-I subdomain separates the more widely conserved SET-N and SET-C subdomains. *B,* surface representation of the three-dimensional structure of the MLL1 SET domain (PDB code 2W5Z (12)). Histone H3 peptide is indicated with carbon atoms in *white*, nitrogen atoms in *blue*, and oxygen atoms in *red*. The co-factor product AdoHcy is indicated with carbon atoms in *green*, nitrogen atoms in *blue*, oxygen atoms in *red*, and sulfur atoms in *yellow*. The position of cysteine 3882 is indicated and highlighted in *yellow. C,* blow-up of the *dashed box* in *B* shows that the Cys-3882 side chain sulfur is greater than 10 Å away from the predicted position of the AdoMet sulfonium moiety. *D,* superposition of Cα atoms of the SET domains from MLL1 (*magenta,* PDB code 2W5Z (12)) and Dim5 (*green,* PDB code 1PEG (30)). The positions of Cys-3882 in MLL1 and the equivalent residue in Dim5 (Val-203) are indicated.

**FIGURE 6.**
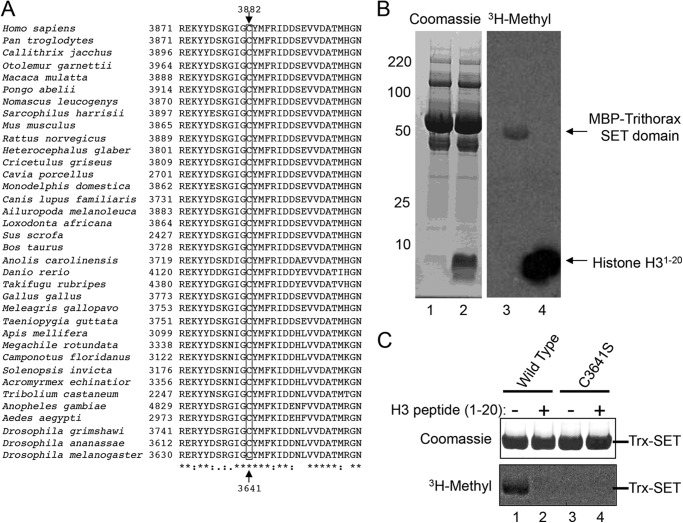
**SET domain automethylation is conserved throughout metazoan evolution.** *A,* ClustalW2 multiple sequence alignment of SET-I subdomain amino acids among metazoan MLL1 orthologs. The position of Cys-3882 in MLL1 (*Homo sapiens* numbering) is *boxed* and indicated at the top. The equivalent position (Cys-3641) in the *D. melanogaster* Trithorax protein is indicated at the *bottom. B,* SET domain from the *D. melanogaster* Trithorax protein was purified as an MBP fusion and incubated with [^3^H]AdoMet in the presence (*lanes 2* and *4*) and absence (*lanes 1* and *3*) of histone H3 peptide (residues 1–20). The Coomassie Blue-stained gel is shown on the *left* and fluorogram on the *right. C,* comparison of automethylation activities of wild type and C3641S Trithorax SET domains in the presence and absence of histone H3 peptide.

Cys-3882 is located in the SET-I region of the MLL1 SET domain (Fig. 5*A*), a region that is less conserved between SET domain families but highly conserved among SET1 family enzymes. Cys-3882 is conserved in all metazoan MLL1 orthologs (Fig. 6*A*). The SET-I region separates two canonical subdomains that are more highly conserved in all SET domain proteins, the SET-N and SET-C regions. The recent crystal structure of the MLL1 SET domain (12) reveals that histone H3 binds in a deep groove within the protein that is flanked by two acidic lobes, one of which is composed of residues from the SET-I subdomain and the other with residues from the SET-C and post-SET subdomains (Fig. 5*B*). Cys-3882 is located in the SET-I lobe ∼10 Å away from the putative position of the sulfonium group of AdoMet, which binds to the opposite lobe (Fig. 5*C*). This distance indicates that domain closure is required for MLL1 SET domain automethylation. Indeed, superposition of the MLL1 SET domain ternary complex with that of the ternary complex of the Dim5 SET domain shows that the Cβ atom of valine 203 in Dim5 (the residue equivalent to Cys-3882 in MLL1) is ∼4 Å closer to the sulfonium of AdoMet than that of the Cβ atom of Cys-3882 in MLL1 (Fig. 5*D*). Histone H3 binds in the groove between domains, providing a plausible explanation for why MLL1 SET domain automethylation is reduced in the presence of histone peptides. It is possible that the conformational flexibility of the SET-I subdomain is restricted when histone H3 peptide is bound, which may prevent the domain closure required for Cys-3882 methylation. Alternatively, it is possible that the ϵ-amino group of H3K4 is better aligned for the *S*_n_2 nucleophilic attack on AdoMet when bound to the SET domain.

##### Cys-3882 Automethylation Is Likely Conserved throughout Metazoan Evolution

Amino acid sequence alignments of vertebrate and invertebrate MLL1 orthologs reveal that Cys-3882 is conserved throughout metazoan evolution (Fig. 6*A*). To test the hypothesis that Cys-3882 automethylation is conserved, we cloned the SET domain from the *D. melanogaster* Trithorax protein into an MBP fusion vector and purified the protein after overexpression in *E. coli*. Enzymatic assays reveal that the MBP-Trithorax SET domain is enzymatically active and undergoes automethylation in the absence of histone H3 (Fig. 6*B*, *lane 3*). Like MLL1, automethylation is reduced in the MBP-Trithorax SET domain in the presence of excess histone H3 peptide (Fig. 6*B*, *lane 4*). To determine whether the residue equivalent to MLL1 Cys-3882 in Trithorax (Cys-3641) is the site of automethylation, we substituted Cys-3641 with serine and tested for automethylation activity in the presence and absence of histone H3. As shown in Fig. 6*C*, replacement of Cys-3641 in the Trithorax protein abolishes SET domain automethylation. These results suggest that automethylation may be a conserved feature of MLL1 orthologs throughout metazoan evolution.

##### MLL1 Automethylation Is Reduced upon Interaction with WRAD

**FIGURE 7.**
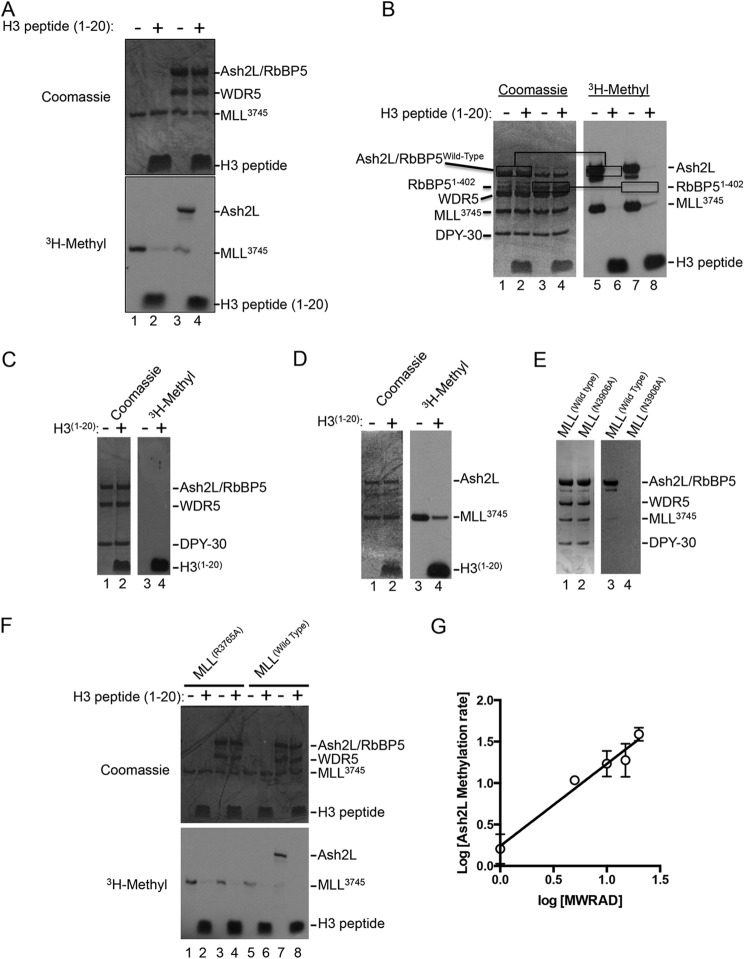
**MLL1 automethylation is reduced when assembled into the MLL1 core complex.** *A,* automethylation of MLL^3745^ in the absence (*lanes 1* and *2*) or presence (*lanes 3* and *4*) of the WDR5-RbBP5-Ash2L subcomplex. Enzymes (7 μm) were incubated with 1 μm [^3^H]AdoMet in the presence or absence of 300 μm histone H3 peptide for 8 h. Quenched samples were separated by 4–12% PAGE and visualized by Coomassie Blue staining (*upper panel*) and fluorography (*lower panel*). *B,* comparison of automethylation activities of MWRAD complexes assembled with a full-length (residues 1–538) or truncated form of RbBP5 (residues 1–402). Assays were carried out as described above. The positions of Ash2L and RbBP5 are indicated with the *boxes. C,* Ash2L is not methylated in the context of WRAD in the absence of MLL1. Assays were visualized by fluorography as described above. *D,* MLL1 does not methylate Ash2L in the absence of WDR5 and RbBP5. *E,* Ash2L methylation depends on the catalytic activity of the MLL1 SET domain. Ash2L methylation was compared between MWRAD complexes assembled with wild type (*lanes 1* and *3*) or N3906A (*lanes 2* and *4*) MLL^3745^ SET domains in the absence of histone H3. *Lanes 1* and *2* are Coomassie Blue-stained gel, and *lanes 3* and *4* are the fluorogram of the same gel. *F,* Ash2L methylation depends on the interaction of MLL1 with WRAD. Wild type (*lanes 5–8*) and R3765A (*lanes 1–4*) variants of MLL^3745^ were assayed in the presence and absence of a stoichiometric amount of WRAD and 300 μm histone H3 peptide for 8 h as described above. *G,* log-log plot showing the concentration dependence of Ash2L methylation within the context of the MLL1 core complex (MWRAD). The data were fit with linear regression showing a slope of 0.99 ± 0.1 (*R*^2^ = 0.93). *Error bars* represent one standard deviation from the mean from two independent experiments.

To determine whether WRAD affects the automethylation activity of MLL1, we compared incorporation of [^3^H]methyl into the SET domain of MLL1 in the presence and absence of WRAD and a histone H3 peptide by fluorography. As shown in Fig. 7*A*, in the absence of histone H3, MLL1 automethylation is reduced by the addition of WRAD (compare *lanes 1* and *3*). Surprisingly, we observed a methylation band corresponding in size to Ash2L or RbBP5 (Fig. 7*A, lane 3*). Upon addition of a stoichiometric excess of histone H3, both bands disappear (Fig. 7*A, lane 4*). These results suggest that WRAD alters the conformational dynamics of the MLL1 SET domain in the complex such that MLL1 automethylation is reduced. Alternatively, MLL1 automethylation may be reduced in the complex due to competition for methylation of Ash2L (see below).

##### Ash2L Is Methylated by MLL1 within the Context of the MLL1 Core Complex

To ascertain whether RbBP5 or Ash2L is methylated within the MLL1 core complex, we compared automethylation activities of complexes assembled with wild type RbBP5 (residues 1–535) or a C-terminal truncated form of RbBP5 consisting of amino acid residues 1–402 (RbBP5(1–402)). If RbBP5 is methylated within the MLL1 core complex, then we expected the position of the radiolabeled band to be altered on the fluorogram when the complex assembled with RbBP5(1–402) was incubated with [^3^H]AdoMet. However, the results show that the pattern of methylation is unchanged with the complex assembled with RbBP5(1–402) compared with that of wild type RbBP5 (Fig. 7*B*, compare *lanes 1* and *5* and *lanes 3* and *7*), indicating that it is Ash2L that is methylated within the MLL1 core complex in the absence of histone H3. To determine whether Ash2L is methylated in the absence of MLL1, we incubated WRAD with [^3^H]AdoMet in the presence and absence of histone H3 peptides. As shown in Fig. 7*C*, despite significant histone H3 methylation by WRAD, no Ash2L methylation was observed in the presence or absence of histone H3. To determine whether the MLL1 SET domain catalyzes Ash2L methylation in the absence of WDR5 and RbBP5, we incubated MLL^3745^ with Ash2L in the presence of [^3^H]AdoMet. Despite observing significant histone H3 methylation and MLL1 automethylation, no Ash2L methylation was observed (Fig. 7*D*).

Together, these results suggest that Ash2L methylation depends on the catalytic activity of the MLL1 SET domain, but only within the context of the MLL1 core complex. To test this hypothesis, we compared Ash2L methylation between MLL1 core complexes assembled with wild type or the catalytically inactive N3906A MLL1 variant. We previously demonstrated that the N3906A variant assembles into the core complex with a sedimentation coefficient similar to that of wild type MLL1 (5). As shown in Fig. 7*E*, Ash2L methylation is absent in the complex assembled with the catalytically inactive variant of MLL1, indicating that Ash2L methylation is dependent on the catalytic activity of the MLL1 SET domain.

To further test the hypothesis that Ash2L methylation occurs only within the context of the MLL1 core complex, we mixed WRA (the minimal complex required for WRAD activity (14)) with a variant of MLL1 containing the R3765A substitution, which was previously shown to abolish the interaction of MLL1 and WRAD, resulting in the loss of H3K4 dimethylation activity of the MLL1 core complex (15, 21–23). As shown in Fig. 7*F*, the R3765A variant of MLL1 is capable of automethylation and methylation of histone H3 (*lanes 1* and *2*), similar to that of wild type MLL1 (*lanes 5* and *6*). However, unlike that observed with wild type MLL1, when the R3765A variant is mixed with WRA, no Ash2L methylation was observed (Fig. 7*F*, compare *lanes 3* and *7*). These results indicate that Ash2L is methylated by MLL1 only within the context of the fully assembled MLL1 core complex.

To determine whether Ash2L methylation occurs in an intra- or intermolecular fashion, we examined the concentration dependence of Ash2L methylation within the context of the MLL1 core complex (MWRAD). We measured the rate of Ash2L methylation as a function of time and MWRAD concentration. The logarithmic plot of the rate of Ash2L methylation as a function of MWRAD concentration shows a slope of ∼0.99 (Fig. 7*G*), which is consistent with a first order reaction mechanism and intramolecular methylation.

Taken together, these results suggest that in the absence of histone H3, the core complex is assembled such that sequences from Ash2L interact with the SET domain as a substrate for methylation by MLL1 in an intramolecular (intra-complex) manner. This would explain why both Ash2L methylation and MLL1 automethylation reactions are inhibited by the addition of unmethylated histone H3. Further studies will be required to identify sites of methylation in Ash2L and their functional significance.

##### Pattern of Inhibition of MLL1 and Ash2L Automethylation Reactions Is Consistent with the Two-active Site model for Multiple H3K4 Methylation

The one-active site model for multiple lysine methylation by the MLL1 core complex predicts that unmodified, mono-, and dimethylated histone H3 species will bind as substrates to the MLL1 SET domain active site and inhibit MLL1 automethylation and Ash2L methylation reactions. However, in the two-active site model, although the unmodified form of histone H3 is predicted to bind to the SET domain and inhibit these reactions, mono-, di-, or trimethylated histone H3 species are not expected to bind to the SET domain as substrates and are therefore not expected to inhibit MLL1-catalyzed automethylation. To distinguish these models, we compared MLL1-catalyzed automethylation activities within the context of the MLL1 core complex in the presence of full-length histone H3 species that were either unmodified or previously mono-, di-, or trimethylated at lysine 4 (as MLA). Sub-stoichiometric amounts of histones were used in the assays to reduce the possibility that inhibition of automethylation may be due to product binding to the SET domain.

**FIGURE 8.**
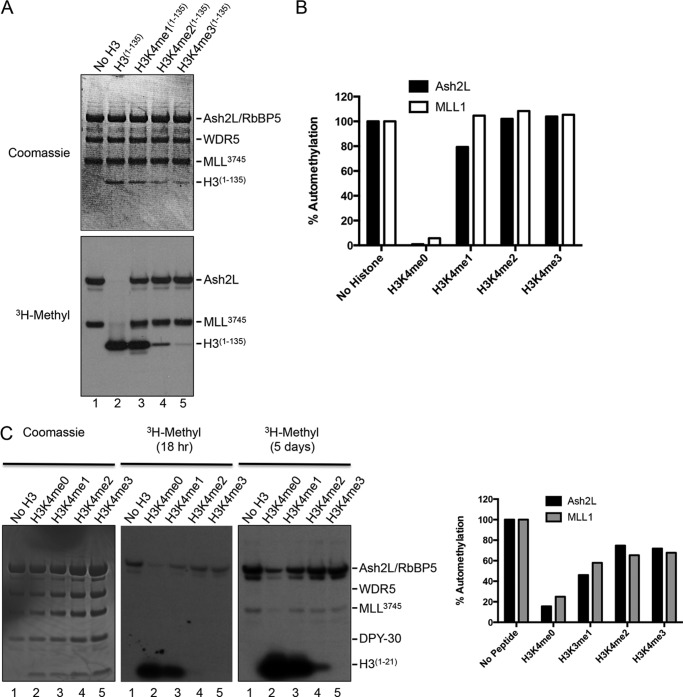
**MLL1 and Ash2L automethylation reactions distinguish one- *versus* two-active site models for multiple H3K4 methylation.** *A,* comparison of MLL1 and Ash2L automethylation activities in the absence (*lane 1*) or presence (*lanes 2–5*) of full-length recombinant histone H3 proteins that were unmodified or previously mono-, di-, or trimethylated at lysine 4 as MLA (from Active Motif). The *upper panel* shows the Coomassie Blue-stained gel, and the *lower panel* is the fluorogram of the same gel. *B,* ImageJ densitometry of the fluorogram in *A*. The *vertical axis* shows percent intensity of each band relative to the intensity of Ash2L or MLL1 methylation in Fig. 8*A*, *lane 1*. Histone H3 bands were saturated at this exposure and were not included in the densitometry. *C,* comparison of MLL1 and Ash2L automethylation activities in the presence or absence of histone H3 peptides (residues 1–21, purchased from Millipore) that were unmodified or previously mono-, di-, or trimethylated at lysine 4. The *left-most panel* shows the Coomassie Blue-stained gel; the *middle panel* shows fluorogram after an overnight exposure (18 h), and the *right panel* shows the fluorogram of the same gel after a 5-day exposure. On the *right* is ImageJ densitometry of the fluorograms showing amounts of Ash2L or MLL1 methylation relative to their respective intensities in the absence of histone peptide (*1st lane*).

When the unmodified H3 protein is incubated with MLL1 and WRA in the presence of [^3^H]AdoMet, both MLL1 automethylation and Ash2L methylation activities are significantly reduced (Fig. 8*A*, *lane 2*). The relative intensity of the Ash2L and MLL1 bands is reduced 99 and 95%, respectively, when in the presence of unmodified histone H3 compared with those same bands in the absence of histone H3 (Fig. 8*B*). However, despite being a robust substrate for di-methylation, the H3K4 monomethylated species does not significantly inhibit MLL1 catalyzed automethylation reactions when incubated with the MLL1 core complex (Fig. 8*A*, *lane 3*). Similarly, inhibition of automethylation was not observed when previously di- and trimethylated histone H3 MLA proteins were incubated with the MLL1 core complex (Fig. 8*A*, *lanes 4* and *5*). A similar pattern of inhibition was observed, albeit to a lesser extent, when stoichiometric amounts of histone H3 peptides were used in the assays instead of full-length histone H3 (Fig. 8*C*). These results are consistent with the predictions of the two-active site model for multiple lysine methylation.

We note that H3K4me2/3 MLA histones show small amounts of methylation at this exposure (Fig. 8*A*, *lanes 4* and *5*), likely due in part to incomplete removal of unreacted histones in the chemical alkylation reaction, methylation at another site in histone H3, or in the case of the H3K4me2 protein, *bona fide* H3K4 trimethylation. We note that trace amounts of H3K4 trimethylation have been observed in quantitative MALDI-TOF assays with the MLL1 core complex (5), but it represent <5% of total methylation, consistent with the MLL1 core complex being predominantly an H3K4 dimethyltransferase.

##### WRAD Preferentially Catalyzes H3K4 Dimethylation within the MLL1 Core Complex

**FIGURE 9.**
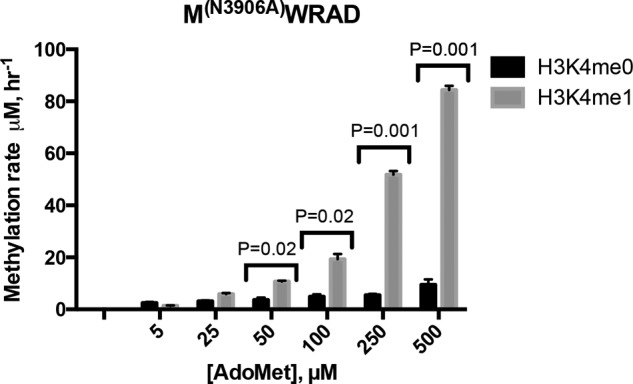
**WRAD preferentially utilizes the H3K4me1 substrate when assembled with the catalytically inactive N3906A MLL1 variant.** The MLL1^(N3906A)^ variant was assembled with WRAD, and the complex was purified by size-exclusion chromatography (Superdex 200, GE Healthcare). Complex was assayed with different concentrations of [^3^H]AdoMet (0–500 μm) with a fixed concentration of unmodified histone H3 peptide (500 μm) (residues 1–20, H3K4me0) or 500 μm H3K4me1 peptide. Samples were quenched at various time points with 1× SDS loading buffer and separated by 4–12% SDS-PAGE. Histone H3 peptide bands were excised from the gel and quantitated by liquid scintillation counting as described under “Experimental Procedures.” *Error bars* represent the standard error of measurement from two independent experiments. The unpaired Student's *t* test in Prism (GraphPad) was used to test for statistical significance. *p* values < 0.05 are indicated.

The results presented above suggest that the H3K4me1 species binds to WRAD and not the MLL1 SET domain during the dimethylation reaction. However, these results do not rule out the possibility that a conformational rearrangement of the MLL1 SET domain allows both automethylation and H3K4 dimethylation activities to occur in the same active site. To distinguish these hypotheses, we compared the enzymatic activity of WRAD in the presence of the catalytically inactive N3906A MLL1 SET domain variant (M^(N3906A)^WRAD) using either unmodified H3K4 (H3K4me0) or H3K4me1 peptides (both at 500 μm) as substrates. When the assays were conducted with low concentrations of AdoMet (0–25 μm), both peptides were methylated but with little difference in activity between H3K4me0 or H3K4me1 substrates (Fig. 9). In contrast, at higher concentrations of AdoMet (>25 μm), the H3K4me1 substrate showed an AdoMet-dependent increase in activity, with little change in the activity with the H3K4me0 substrate. For example, at a concentration of 500 μm AdoMet, activity with the H3K4me1 substrate was almost an order of magnitude greater than that with an equivalent concentration of the H3K4me0 substrate (Fig. 9). These results are consistent with the hypothesis that WRAD preferentially catalyzes H3K4 dimethylation within the MLL1 core complex.

## DISCUSSION

### 

#### 

##### Multiple Methylation and the SET Domain

SET domain enzymes that catalyze multiple methylation events invariably contain a phenylalanine at the key Phe/Tyr switch position in their active sites (24). In contrast, SET domain enzymes that possess a tyrosine in the switch position have smaller active site volumes and are monomethyltransferases. The MLL1 SET domain contains a tyrosine in the switch position and is therefore predicted to be a monomethyltransferase. However, a purified MLL1-containing super-complex has been shown to possess H3K4 mono-, di-, and trimethyltransferase activity (25). This paradox has led to the suggestion that proteins that interact with the MLL1 SET domain allosterically alter the orientation of the tyrosine in the switch position to regulate the degree of SET domain-catalyzed H3K4 methylation (26). This “one-active site” model is based on the assumption that the MLL1 SET domain is the only known methyltransferase motif in the complex. Often cited in support of this assumption is the claim that all H3K4 methylation is lost in *Saccharomyces cerevisiae* when the gene for the MLL1 homolog SET1p is deleted (10, 18, 19, 27). However, these early studies were based on results obtained from Western blots using antibodies raised against the H3K4me2 or H3K4me3 epitopes and therefore do not exclude the possibility that H3K4me1 may still be present. Indeed, there is evidence for weak H3K4 monomethylation activity in *S. cerevisiae* SET1 knock-out strains (28). SET1p assembles into a multisubunit complex called COMPASS with subunits homologous to those found in the MLL1 core complex (18). What is lacking is an unambiguous demonstration that the SET domain is responsible for the observed multiple methylation behavior of these super-complexes.

Several groups have attempted to address this issue with biochemical reconstitution systems. Roeder and co-workers (6) attempted a biochemical reconstitution of human MLL1 with its core components WDR5, RbBP5, and Ash2L using a baculovirus co-expression and immunopurification system from insect cells. They observed by Western blotting multiple H3K4 methylation (mono-, di-, and possibly trimethylation) with their immunopurified MLL1 core complex but did not conclusively show that the MLL1 subunit was responsible for multiple methylation. Wilson and co-workers (12) determined the atomic structure of the MLL1 SET domain and noted that it was different from the structures of other SET domain proteins in that it was locked in an inactive open conformation in the presence or absence of ligands. They demonstrated a 20-fold stimulation of activity by addition of WRAD to the same MLL1 construct and concluded that protein interaction-induced conformational changes are required for closing the SET domain for full activity (12). However, the work reported here describes the discovery of MLL1 automethylation activity at an evolutionarily conserved cysteine residue in the SET domain active site. The location of Cys-3882 in the SET-I subdomain, more than 10 Å from the putative position of the sulfonium group of AdoMet in the crystal structure, indicates that domain closure is necessary for automethylation. The observation that MLL1 automethylation reaction is relatively robust in the absence of histone H3 implies that the SET domain possesses significantly more conformational plasticity in solution than suggested by its crystal structure. Furthermore, the MLL1 SET domain construct utilized by Wilson and co-workers (12) did not contain the evolutionarily conserved Win motif, which has been shown to be required for MLL1 core complex formation (15, 21). This observation, along with the recent discovery that WRAD possesses enzymatic activity on its own (5, 14), makes it difficult to determine how much MLL1 SET domain activity is stimulated by WRAD.

##### Two Active-site Model for Multiple Methylation

Several lines of evidence suggest that the MLL1 core complex uses two distinct active sites to catalyze multiple methylation of H3K4. We previously demonstrated using quantitative MALDI-TOF mass spectrometry-based methylation assays that the MLL1 SET domain is an intrinsic H3K4 monomethyltransferase (5), consistent with the predictions of the Phe/Tyr-switch hypothesis (29, 30). This activity was shown to be dependent on Tyr-3942 of MLL1, as replacement with Phe converts MLL1 into a processive trimethyltransferase (5). In contrast, single turnover enzyme kinetics experiments reveal that the core complex assembled with wild type MLL1 is a nonprocessive dimethyltransferase, as there is a clear accumulation of a monomethylated intermediate during the course of the reaction (5). These data suggest that histone H3 dissociates from the first active site after the addition of the first methyl group. Monomethyl H3K4 may then either rebind the MLL1 SET domain or bind to a different active site to undergo the dimethylation reaction.

Lack of inhibition of automethylation by H3K4me1 substrate, despite being a robust substrate for dimethylation, suggests that the H3K4me1 intermediate does not bind to the canonical SET domain during the dimethylation reaction. However, lack of automethylation inhibition by itself does not rule out the possibility of a more complex conformational rearrangement of the SET domain that allows both automethylation and H3K4 dimethylation reactions to occur simultaneously in the same active site. The fact that the H3K4 dimethylation reaction can be reconstituted with a complex containing a SET domain variant that lacks catalytic activity (5) argues strongly in favor of a two active-site model for multiple methylation. The two active-site model is supported by the observation that WRAD possesses intrinsic H3K4 monomethyltransferase activity in the absence of MLL1 (5, 14).

**FIGURE 10.**
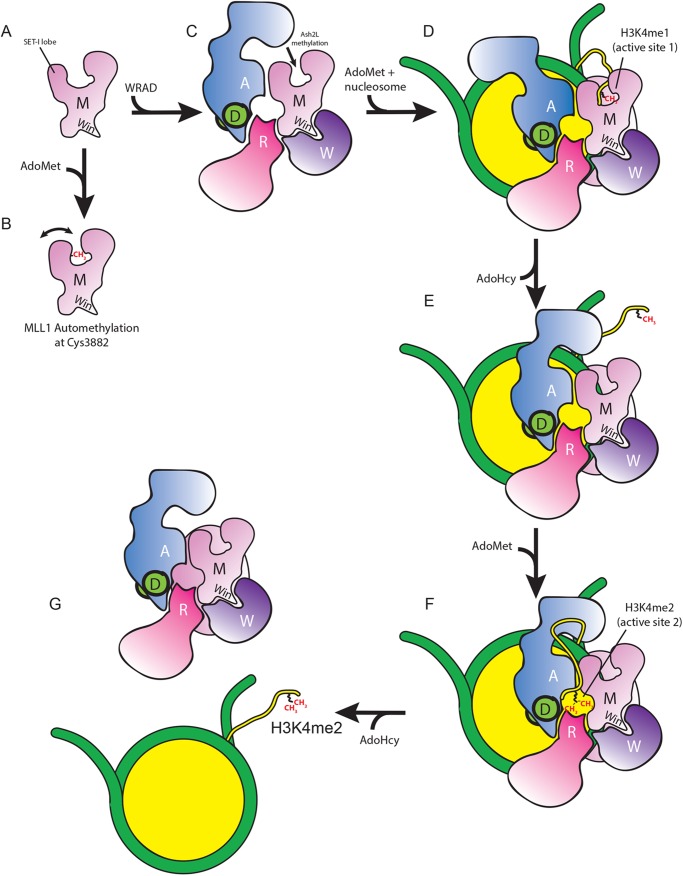
**Model for multiple lysine methylation by MLL1 core complex.** *A,* MLL1 SET domain (*M*) is shown in *pink* with the SET-I lobe and Win motif indicated. *B,* MLL1 undergoes automethylation at Cys-3882 in the presence of AdoMet and absence of histone H3. *C,* WRAD binding to the Win motif of MLL1 positions N-terminal sequences of Ash2L (*A*) near the SET domain of MLL1. In the absence of histone H3, Ash2L can then be methylated by MLL1. *D,* nucleosome (*yellow/green*) binding to the MLL1 core complex positions the histone H3 N-terminal tail in the MLL1 SET domain active site where it undergoes monomethylation at lysine 4 (H3K4me1). *E,* H3K4me1 intermediate diffuses away from the MLL1 SET domain, rendering it accessible for automethylation or Ash2L methylation activities. *F,* H3K4me1 intermediate undergoes dimethylation at active site 2, which is composed of sequences from WRAD and a surface from MLL1 that is distinct from the canonical SET domain active site. *G,* the MLL1 core complex dissociates from the nucleosome.

The most parsimonious model that accounts for all of the available data is the one in which two distinct active sites catalyze each methylation event in a stepwise manner (Fig. 10). In this model, we propose that the first methylation event is catalyzed by the MLL1 SET domain, which is supported by the observation that stoichiometric amounts of unmodified but not mono-, di-, or trimethylated species of H3K4 inhibit the MLL1 automethylation reaction. We note MLL1 automethylation is inhibited by excess H3K4me1 (but not by excess H3K4me2/3 (data not shown)), likely due to binding to the SET domain as a product. The H3K4me1 intermediate then dissociates from the SET domain active site and binds to the second active site composed of sequences from WRAD and MLL1 to undergo dimethylation. Such a mechanism is reminiscent of several multifunctional enzymes that employ a “swinging arm” to deliver a substrate to successive active sites in multistep reactions (for a review see Ref. 31). Given the intrinsic flexibility of the histone H3 N-terminal tail, it is possible it could serve as a tethered swinging arm that delivers the H3K4 substrate to each active site.

Although the two-active site model accounts for the kinetic and automethylation behavior of the MLL1 core complex, there are still some ambiguities that need to be addressed. Although WRAD activity has now been observed by other groups (32, 33), it has been suggested that its activity is too low to be physiologically relevant (33). However, a careful steady-state kinetic analysis where stoichiometric complex was assayed reveals that human WRAD has an apparent *k*_cat_ value of ∼30 h^−1^ (14), which is similar to that of other histone methyltransferases, with *k*_cat_ values that range from 12 to 396 h^−1^ (34).

Another question is why WRAD requires MLL1 (either wild type or catalytically inactive) to catalyze H3K4 dimethylation? This observation suggests that the second active site requires WRAD and a surface from MLL1 that is distinct from the canonical SET domain active site cleft to directly or indirectly form the dimethyltransferase active site (Fig. 10). The identity of this MLL1 surface is suggested by our recent demonstration that a common cluster of amino acids that reside outside of the SET domain active site cleft are required for stabilization of the interaction of RbBP5 and Ash2L with the core complex and for the H3K4 dimethyltransferase activity of the MLL1 core complex.^8^ This same cluster of amino acids was previously shown to be among a spectrum of missense mutations in MLL2 that are associated with a human multiple malformation disorder called Kabuki syndrome (35–38).

The last ambiguity is why WRAD in the context of the core complex requires the H3K4me1 substrate to catalyze H3K4me2? In other words, why does it not use the H3K4me0 substrate to catalyze mono- and dimethylation? We propose that WRAD is a one-carbon methyltransferase enzyme that forms part of a unique binding site when in complex with MLL1 that specifically recognizes the H3K4me1 intermediate. This hypothesis is supported by the data presented in this investigation where we demonstrate that the core complex assembled with a catalytically inactive variant of MLL1 preferentially methylates the H3K4me1 substrate over unmodified H3. In addition, it is possible that when WRAD methylates unmodified H3K4, the ϵ-amino group does not possess the rotational freedom when bound to the enzyme to align the lone pair for a second nucleophilic attack. This model may explain why WRAD in the absence of MLL1 does not have appreciable H3K4 dimethyltransferase activity. In summary, our data suggest that WRAD is an H3K4 monomethyltransferase that preferentially monomethylates the H3K4me1 substrate when assembled into the MLL1 core complex.

##### Possible Physiological Significance of MLL1 Automethylation Activities

The physiological significance of MLL1-catalyzed automethylation reactions is unknown. The fact that Cys-3882 is conserved among vertebrate and invertebrate orthologs of MLL1 and MLL4, but not in unicellular eukaryotes, suggests that it is retained for some aspect of metazoan evolution. In addition, cysteine is not conserved in the same amino acid position in the other human SET1 paralogs MLL2/3 and SETd1a/b (data not shown), suggesting that it is retained for some function that is unique to MLL1/4. AdoMet is metabolically expensive to produce as ∼3 ATP equivalents are expended in its synthesis (39). It therefore seems probable that evolution would have selected against conservation of cysteine at position 3882 of MLL1 if it were not otherwise contributing some kind of selective advantage.

Automodification activities are common among post-translational modification enzymes and have been shown to play diverse roles that include regulation of enzymatic activity, effector protein recruitment, and protein stabilization. Automodifications that regulate enzymatic activity have been observed in kinases (40), histone acetyltransferases (41, 42), and protein and DNA methyltransferases (43–47). For example, automethylation of Lys-485 of the SET domain protein Metnase inhibits its enzymatic activity and represses topoisomerase IIα-catalyzed chromosome decatenation (45). Automethylation of an arginine residue in the C-terminal domain of coactivator-associated arginine methyltransferase-1(CARM1) has been shown to be important for CARM1-associated transcriptional activation and pre-mRNA splicing *in vivo* (44). Automethylation of a conserved lysine residue in the primary sequence of the G9a SET domain enzyme has been shown to be important for its interaction with heterochromatin protein-1 (HP1) (43, 48). However, unlike MLL1 automethylation, G9a, Metnase, and CARM-1 automethylation reactions are intermolecular and result in methylation of lysine (G9a and Metnase) or arginine (CARM-1) residues that reside outside of their catalytic domains and mimic their cognate substrates. Because Ash2L contains several H3K4-like sequences, it is possible that Ash2L methylation within the MLL1 core complex serves a similar role *in vivo*.

##### Cysteine Methylation in Biology

Cysteine methylation or *S*-methylation has been detected in proteins with diverse functions, including hemoglobin (49), human lens crystallins (50), DNA methylation/repair enzymes (46, 51, 52), and in proteins involved in regulation of NF-κB signaling (53). Crystallin *S*-methylation has been suggested to serve a protective role from cataractogenesis by preventing intermolecular disulfide bond formation and protein aggregation (50). In contrast, automethylation of the catalytic cysteine residues in bacterial (BspRI and Dcm-C5 (51, 54)) and mammalian (Dnmt3a (46)) DNA methyltransferases in the absence of DNA inhibits catalytic activity and has been suggested to either play a regulatory role in response to changes in cellular AdoMet/AdoHcy concentrations or to be an aberrant side reaction due to the high methyl group transfer potential of AdoMet (46). Given that the apparent *K_m_* values for AdoMet in the MLL1 automethylation and histone H3 methylation reactions are similar to that of other AdoMet-dependent enzymes (55) and in the range of intracellular AdoMet concentrations (estimated at ∼30 μmol/liter in non-liver tissue (55)), it is possible that MLL1 *S*-methylation could play a similar regulatory role in response to changes in the AdoMet/AdoHcy ratio.

More recently, a novel role for *S*-methylation has been suggested for regulating the ubiquitin chain-sensing functions of TGF-β-activated kinase-binding proteins TAB-2/3 in the NF-kB signaling pathway in response to bacterial infection (53). It has been demonstrated that the enteropathogenic *E. coli* protein NleE possesses an AdoMet-dependent cysteine methyltransferase activity that specifically modifies a zinc-coordinating cysteine residue in TAB2/3, which abolishes their ubiquitin chain binding activity (53). Because MLL1 requires zinc for catalytic activity, it is possible that *S*-methylation could play a similar role in regulating the H3K4 methylation by MLL1. However, automethylation of MLL1 involves a cysteine residue (Cys-3882) that does not coordinate zinc in the crystal structure (12). In addition, mutation of Cys-3882 to alanine or serine does not alter enzymatic activity. Therefore, it is likely that the automethylation at Cys-3882 in MLL1 serves a different function.

The last example includes a novel form of *S*-methylation that involves enzymatic methyl group transfer to proteins that does not involve AdoMet as a methyl donor. This mechanism is central to bacterial and mammalian DNA damage repair enzymes that remove a methyl group from *O*^6^-methylguanine (56, 57). In these enzymes, an active site cysteine acts as a nucleophile to accept a methyl group from the *O*^6^ position in an apparent suicide reaction that repairs DNA alkylation damage. Although the bacterial Ada enzyme repairs *O*^6^-methylguanine in this manner, it also repairs DNA methylphosphotriesters by transfer of the methyl group to another cysteine residue in the protein, which induces a conformational change that allows it to bind DNA and activate transcription (58–60).

These studies illustrate the diverse functional consequences of *S*-methylation in cells. Further studies will be required to understand the role of *S*-methylation in MLL1 biology.

## Footnotes

8 S. A. Shinsky and M. S. Cosgrove, submitted for publication.

## Footnotes

7 The abbreviations used are:

MBP: maltose-binding protein
AdoMet: *S*-adenosylmethionine
AdoHcy: *S*-adenosylhomocysteine
BisTris: 2-[bis(2-hydroxyethyl)amino]-2-(hydroxymethyl)propane-1,3-diol
MLA: methylated lysine analog
PDB: Protein Data Bank
ESI: electrospray ionization.
